# Optimising diagnosis of viraemic hepatitis C infection: the development of a target product profile

**DOI:** 10.1186/s12879-017-2770-5

**Published:** 2017-11-01

**Authors:** Elena Ivanova Reipold, Philippa Easterbrook, Alessandra Trianni, Nivedha Panneer, Douglas Krakower, Stefano Ongarello, Teri Roberts, Veronica Miller, Claudia Denkinger

**Affiliations:** 1 FIND, MSF Access Campaign, Geneva, Switzerland; 2 Forum for Collaborative HIV Research, Washington D.C, USA; 3 Beth Israel Deaconess Medical Center, Harvard Medical School, Boston, MA USA; 4 Global Hepatitis Programme, HIV Department, World Health Organization, Geneva, Switzerland

## Abstract

**Background:**

The current low access to virological testing to confirm chronic viraemic HCV infection in low- and middle-income countries (LMIC) is limiting the rollout of hepatitis C (HCV) care. Existing tests are complex, costly and require sophisticated laboratory infrastructure. Diagnostic manufacturers need guidance on the optimal characteristics a virological test needs to have to ensure the greatest impact on HCV diagnosis and treatment in LMIC. Our objective was to develop a target product profile (TPP) for diagnosis of HCV viraemia using a global stakeholder consensus-based approach.

**Methods:**

Based on the standardised process established to develop consensus-based TPPs, we followed five key steps. (i) Identifying key potential global stakeholders for consultation and input into the TPP development process. (ii) Informal priority-setting exercise with key experts to identify the needs that should be the highest priority for the TPP development; (iii) Defining the key TPP domains (scope, performance and operational characteristics and price). (iv) Delphi-like process with larger group of key stakeholder to facilitate feedback on the key TPP criteria and consensus building based on pre-defined consensus criteria. (v) A final consensus-gathering meeting for discussions around disputed criteria. A complementary values and preferences survey helped to assess trade-offs between different key characteristics.

**Results:**

The following key attributes for the TPP for a test to confirm HCV viraemic infection were identified: The scope defined is for both HCV detection as well as confirmation of cure. The timeline of development for tests envisioned in the TPP is 5 years. The test should be developed for use by health-care workers or laboratory technicians with limited training in countries with a medium to high prevalence of HCV (1.5–3.5% and >3.5%) and in high-risk populations in low prevalence settings (<1.5%). A clinical sensitivity at a minimum of 90% is considered sufficient (analytical sensitivity of the equivalent of 3000 IU/ml), particularly if the test increases access to testing through an affordable price, increase ease-of-use and feasibility on capillary blood. Polyvalency would be optimal (i.e. ability to test for HIV and others). The only characteristic that full agreement could not be achieved on was the price for a virological test. Discussants felt that to reach the optimal target price substantial trade-offs had to be made (e.g. in regards to sensitivity and integration).

**Conclusion:**

The TPP and V&P survey results define the need for an easy-to-use, low cost test to increase access to diagnosis and linkage to care in LMIC.

**Electronic supplementary material:**

The online version of this article (10.1186/s12879-017-2770-5) contains supplementary material, which is available to authorized users.

## Background

Hepatitis C virus (HCV) causes both acute and chronic infection, and is a leading cause of liver-related mortality worldwide [1]. Spontaneous clearance of acute HCV infection occurs in between 15 and 45% of infected individuals in the absence of treatment [2]. The remaining 55–85% of persons who do not clear HCV within six months are defined as having chronic viraemic infection and require treatment to prevent the substantial liver-related morbidity and mortality. It is estimated that 71 million people are living with chronic viraemic HCV infection [3].

The treatment landscape for HCV has undergone a dramatic transformation in recent years, from the use of complex interferon-based regimens with low cure rates and significant rates of adverse effects, to availability of short all-oral direct acting antiviral (DAA) regimens, which are both well tolerated and offer very high cure rates of >90% across different HCV genotypes after 12 weeks of treatment [4]. There has also been recent expansion of access in low- and middle income countries (LMIC) as a result of the availability of generic DAAs [5–7] resulting in substantial price reductions and price of US $ 100–250 for a 2-drug treatment course is within reach [8, 9].

Despite these opportunities for treatment, a major bottleneck to appropriate HCV care is diagnosis [10]. It is estimated that around 20% of infected persons globally have been tested and are aware of their diagnosis [3], but this is much lower in LMICs [11]. There are several key reasons for this low uptake: lack of awareness about HCV among population, exiting diagnostic algorithms are complex, laboratory capacity to diagnose hepatitis C in LMIC remains very low and is largely located in the private sector, and available tests are costly [12–14]. The current diagnostic algorithm first requires screening for evidence of past or current HCV infection with a serological assay that detects hepatitis C antibody (either an enzyme immunoassay (EIA) or by a rapid diagnostic test (RDT)). Those with positive serology will need further testing to confirm the presence of HCV viraemia, and need for treatment, by nucleic acid testing (NAT) detection HCV RNA (either quantitative or qualitative) or antigen detection targeting HCV (p22) core antigen (HCV cAg) [15–17].

**Fig. 1:**
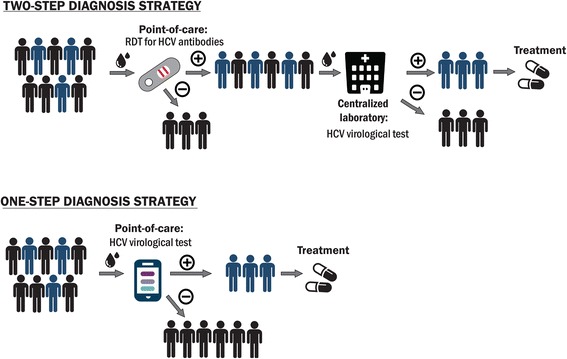
One-step and two-step testing strategy for HCV diagnosis. RDT states for rapid diagnostic test; POC – point-of-care (i.e. test can be performed at any level of patient care)

Currently available commercial quantitative and qualitative HCV RNA or HCV cAg assays are focused on centralized laboratory infrastructure (e.g. Roche COBAS Taqman HCV assay, Abbott RealTime HCV assay, Hologic Aptima® HCV Quant Dx Assay) [13]. While such platforms may play an important role in countries that favour a centralized infrastructure, and have the appropriate specimen referral system in place, improvements are necessary on the specimen collection and transport (e.g. through Dried Blood Spots) and on reaching affordability. Recently, one integrated platform for decentralized use has entered the market (i.e. Cepheid GeneXpert) and has also received WHO pre-qualification [18]. Other promising diagnostics are under development, but manufacturers need further guidance on the optimal characteristics of the tests that can be implemented in LMIC. An HCV RNA or HCV cAg test that can be performed near or at the site of patient care (point-of-care [POC] testing) may help increase access to HCV care especially in resource-limited settings and hard to reach populations. Also, the advantages of rapid turn-around times offered by near-patient or POC platforms may reduce loss to follow-up and allow for immediate treatment decisions [19]. In addition, if these tests can be made affordable, it may be possible to dispense with the need for an initial serological screening test and have a single test strategy to diagnose viraemic infection (Fig. 1).

A target product profile (TPP) is a tool that has been used widely to support and guide drug, and diagnostic development in various fields of health [20, 21]. This article describes the development of a TPP for a near-patient test for diagnosis of HCV viraemia using a global stakeholder consensus-based approach. The TPP process was complemented by a comprehensive survey of values and preferences (V&P survey) to seek additional perspectives on the TPP criteria and capture acceptable trade-offs between test price, performance and operational characteristics. This TPP is intended to inform test developers as well as donors and provide a tool for communication in accelerating the path towards HCV diagnostic tests suitable for scale-up of HCV care in LMIC.

## Methods

### Developing target product profiles for drugs and diagnostics

A target product profile (TPP) is a strategic planning tool – usually in the form of a document that provides a clear statement of desired outcome of product development [22]. There have been several TPPs for diagnosis of tuberculosis, sexually transmitted diseases and others [21, 23–25]. A TPP aims to (i) Define the medical/public health need; (ii) Make the need transparent to test developers to increase interest in the field by defining features and characteristics of the desired test; and (iii) Outline features that would provide a competitive advantage, utilizing the input of key stakeholders in the field. The standard categories of criteria included in a TPP are scope, performance and operational characteristics and price.

### Methodology for development of TPPs for HCV RNA and core antigen tests

There were five key steps in the process of developing this TPP.

1. *Identifying key potential global stakeholders for consultation and input into the TPP development process.* A list of 50 potential stakeholders were identified for consultation and input into the TPP development process. The stakeholders were chosen to provide a representation from (i) National Hepatitis programs; (ii) Implementers and Clinicians (including from NGOs); (iii) Technical agencies and researchers; (iv) International organizations; (v) Advocacy organizations/Civil society; (vi) industry (pharmaceutical and IVD-manufacturers)
2. *Informal priority-setting exercise with key experts.* Second, we undertook an informal priority-setting exercise with key stakeholders to identify the needs that should be the highest priority in the TPP development. Given the availability of centralized platforms to diagnose viraemic infection, it was decided to focus this TPP development on point-of-care or near-patient diagnostic tests that detect the presence of HCV RNA or HCV cAg. Such diagnostic tests were considered to play an important role in settings with challenges in specimen and results transfer and high loss-to-follow-up after initial diagnosis [26, 27].
3. *Defining the key TPP domains (scope, performance and operational characteristics and price).* Third, we defined the domains and characteristics of a comprehensive TPP. The domains included (i) Scope (characteristics: goal of the test, target population, level of the health-care system where the test could be run and intended end user(s) and lowest setting for implementation); (ii) Performance (characteristics: clinical and analytical sensitivity and specificity, polyvalency and quantitation); (iii) Operational (characteristics: specimen type and preparation steps, time to result, specimen through-put and capacity, biosafety and waste disposal, instrumentation, power requirements, maintenance calibration, data analysis, connectivity, result capture, operating temperature/humidity, reagent kit transport/storage and stability, internal quality control); (iv) price (characteristics: maximum price per test and for instrumentation), as well as the most important performance and operational characteristics and prices (Table 1). These characteristics were described in terms of both minimal and optimal characteristics, which defined a range, where “minimal” referred to the lowest acceptable output for a characteristic and “optimal” to the ideal target (preferable/ideal). The original TPPs were developed by FIND and the Forum for Collaborative HIV Research (“the Forum”), in collaboration with WHO’s Global Hepatitis Programme. Additional input was sought from experts in the field at the U.S. Center for Disease Control and Prevention (CDC), Médecins Sans Frontières (MSF), the Treatment Action Group (TAG), and WHO’s Prequalification Program.
4. *Delphi-like process with larger group of key stakeholder to facilitate feedback on the key TPP criteria and consensus building based on pre-defined consensus criteria.* Fourth, a Delphi process [28] was used to facilitate anonymous feedback on the key TPP criteria and consensus building. Organizations/individuals that were invited and agreed to participate were asked to provide a statement on their level of agreement with each of the proposed characteristics for each TPP. Agreement was scored on a Likert scale ranging from 1 to 5 (1 = disagree, 2 = mostly disagree, 3 = do not agree or disagree, 4 = mostly agree, 5 = fully agree). Consensus was pre-defined as more than 50% of respondents providing a score of at least four on the Likert scale (as per WHO policy for expert group consensus). Initially, one round was planned followed by an anonymous summary of the experts’ opinion, followed again by a second survey round. However, since predefined consensus for all characteristics was reached after the first round, a second round was not initiated and thus a “Delphi-like” method was used.
5. *A final consensus-gathering meeting for further discussions around disputed characteristics.* A consensus-gathering meeting was convened by FIND and the Forum, in collaboration with WHO, on April 22, 2015, prior to the European Association for the Study of the Liver’s annual meeting in Vienna, Austria. The invitation to the meeting was sent to 50 organizations/individuals who participated in the TPP survey (step 4) and 35 people were able to attend. No financial support was provided to participants at the meeting. Given the high level of consensus in the Delphi-like process, the main purpose of this meeting was to discuss issues on which fewer than 75% of the respondents agreed, or on which a distinct subgroup disagreed, or on which further need for clarification and discussion was considered useful. If any voting was necessary throughout the consensus-gathering meeting, >75% was considered a majority.

**Table 1:** Combined HCV RNA and cAg TPP for an HCV test

Characteristic	Optimal	Minimal	Rationale and evidence
Scope			
Goal of test	The goal of the test is two-fold: 1. To diagnose active viraemic HCV infection (new or reinfection) and provide baseline virological assessment (quantitative or qualitative); 2. To confirm cure upon treatment completion. Ideally, the test would be done with the purpose of initiating treatment within the same clinical encounter or the same day. Not intended for blood screening. The timeline of development for tests envisioned in the TPP is 5 years.	The goal of the test is two-fold: 1. To diagnose active HCV viraemic infection (new or reinfection) and provide baseline virological assessment (qualitative) with the purpose of initiating treatment; 2. To confirm cure upon treatment completion. Not intended for blood screening. The timeline of development for tests envisioned in the TPP is 5 years.	Detection can be performed by HCV RNA test or by HCV cAg detection. Presence of HCV RNA or cAg in a patient is indicative of active HCV infection. Currently, the HCV RNA or cAg test is performed after a positive anti-HCV serological test (i.e. two-step algorithm). Conceivably, provided the prevalence is substantial and the cost of the HCV RNA or cAg test is low, either test could be used in a one-step algorithm.
Target population	Countries with a medium to high prevalence of HCV (1.5–3.5% and >3.5%) High-risk populations in low prevalence settings (<1.5%).		High-risk populations include: persons who inject drugs or have used intranasal drugs (PWID), people living with HIV (PLWH), men who have sex with men (MSM), prisoners, people with tattoos, sex workers, people with frequent contact with the health-care system (i.e. chronically ill) and children born to HCV-infected mothers. In order to achieve the long-term goal of HCV elimination, optimally the test should be performed on all patients in primary care settings, antenatal clinics and in community screening programmes.
Target operator of test	Community workers with minimal training	Health-care workers or laboratory technicians with limited training (i.e. able to operate an integrated test with minimal additional steps)	
Lowest level of setting for implementation (public & private)	Community centres	District hospital (Level II)	
Performance characteristics			
Diagnostic sensitivity*	>99%	90%–95%	Rationale of optimal test: Ideally a test should be as sensitive and specific as available plasma-based HCV RNA tests. A commonly used reference standard is the VERSANT HCV RNA Qualitative Assay, which is FDA-approved for diagnosis of active HCV infection (although the VERSANT HCV RNA Qualitative Assay is being taken off the market, it remains the most analytically sensitive assay and was used as the gold standard in most instances). Rationale of minimal: If a test is easier to implement at lower levels of the health care system without requiring substantial technical expertise or complex laboratory infrastructure, and is less costly, then a compromise can be made on sensitivity. A test with a suboptimal sensitivity of 90–95% with improved operational characteristics was considered acceptable by stakeholders as it would improve rates of diagnosis substantially over what is currently possible. However, no studies or modelling have been done on the minimal acceptable sensitivity and the optimal other characteristics needed by a test for HCV diagnosis to lead to substantial improvement in HCV detection on a population level. Modelling work for TB has provided insights that could potentially be applicable for HCV as well. A model showed that for the WHO Southeast Asia Region a POC biomarker test with a sensitivity of 50% for smear-negative TB, if employed at the most peripheral health-care setting, would result in a similar reduction in TB incidence as a test with 70% sensitivity for smear-negative TB that would be employed at the district level (e.g. Xpert MTB/RIF) [17]. However, the exact trade-off between a lower sensitivity (for smear-negative TB) and an increase in access to testing is setting dependent. Under the minimal scenario, some patients would be incorrectly diagnosed as not having active HCV infection, What impact that would have on patient and provider behaviour is unclear.
Analytical sensitivity (comparison with HCV RNA test reference standard)	200 IU/ml	1000–3000 IU/ml	Among the majority of infected individuals with chronic HCV infection, HCV RNA viral loads are between 10^4^ and 10^7^ IU/ml [13]. In studies of viral dynamics during acute infection, viral loads as low as 3 log IU/ml (or 1000 IU/ml) were seen during the first four months after infection [13]. The optimal LOD of 200 IU/ml should therefore detect most patients (>99%). At a minimum, analytical sensitivity of 1000–3000 IU/ml or 3 fmol cAg/l (current LOD of the Abbott HCV cAg assay), the corresponding clinical sensitivity should be 90–95%. Upper limit of the dynamic range should be equivalent to that of current laboratory-based quantitative HCV RNA tests. An HCV RNA test should be standardized with the WHO International Standard for Hepatitis C Virus RNA, as has been done with current FDA-approved and CE-marked qualitative and quantitative HCV RNA assays. Interestingly, HCV cAg test have been shown to be negative among a portion of untreated individuals with high HCV RNA levels, indicating the likely presence of mutant variants [18]. The limitation of the cAg assay to accurately detect these variants may present a challenge to elimination. Data from patients who relapsed after treatment with peg-interferon and ribavirin therapy indicate that HCV rebounds quickly to high levels (10^3^ IU/ml and greater) within a few weeks after the end of treatment [19]. Early data from DAA-based therapy suggests that an even more rapid relapse would happen (unpublished data; communication with A. Hill). Given the high correlation between HCV cAg and HCV RNA levels, either test would likely be suitable for monitoring virologic response after treatment completion several weeks after completion of therapy.
Diagnostic specificity (comparison with HCV RNA reference standard)*	>99%	>98%	Since the test is a test for detection of active HCV infection, it should be as specific as current commercially available and FDA-approved HCV RNA tests to avoid false positive results.
Analytical specificity – HCV detection	No cross reactivity with endogenous substance and exogenous factors (e.g. HIV-1, HIV-2, HBV, HEV, antimalarials, anti-TB, ART)	No cross reactivity with endogenous substance and exogenous factors (e.g. HIV-1, HIV-2, HBV, HEV, antimalarials, anti-TB, ART)	
Polyvalency	Ability to detect HIV, hepatitis B on the same instrument		
Quantitation	Quantitative	Qualitative	Treatment monitoring is not considered necessary or feasible with novel DAA agents [14], therefore a qualitative test result is preferred. According to stakeholder opinion, a quantitative result would be beneficial as it allows research questions to be investigated; however, it cannot come at an increased cost.
Operational Characteristics			
Specimen type	Capillary whole blood	Venous whole blood or plasma	The emphasis is for the use of capillary whole blood that can diagnose infection in the clinic without requiring additional laboratory equipment such as a bench top centrifuge. The need for phlebotomy to draw venous whole blood would limit the applicability of the test in lower settings of care as per stakeholder opinion. If plasma is to be a specimen type (minimal criteria), the plasma separation step should be integrated into the instrument.
Specimen prep (total steps)	Integrated specimen preparation (including plasma separation if needed); less than 2 steps required (no precision volume control and precision time steps)	Maximally 2 steps (no precision volume control and precision time steps)	Equipment such as a centrifuge or heat block are available only infrequently at level 1 health centres and some district hospitals, and therefore should not be required for novel assays. Expertise to operate a precision pipette is also often lacking [21]. For the detection of cAg, several specimen preparation steps are needed: i) to dissociate antibody-bound cAg; ii) to lyse viral particles and expose cAg; and iii) to inactivate antibody. These should also optimally be integrated with the test of detection.
Time to result	< 15 min	< 60 min	The need for a rapid turn-around time, the possibility for batching and/or random access for testing, and the testing of multiple specimens at the same time are interrelated. The time to result is probably the most important parameter, as extending the wait time for patients will possibly result in loss to follow-up [22, 23]. Most current immune-chromatographic rapid tests produce results within 20 min. The ideal time to result has not been studied and might vary largely between countries and between settings where the patient is tested. But in order to be deployable as a test for POC, the result should be available within the same visit.
Specimen capacity and throughput	Multiple at a time; random access/parallel processing	One at a time (any external reagents should be aliquoted for one time use)	Preferred that one specimen does not occupy the instrument at a time - i.e., random access/parallel analysis. If the platform is multi-analyte, then running different assays should be feasible at the same time.
Biosafety + waste disposal	Mostly simple waste; minimal biosafety waste; no sharps	No need for a biosafety cabinet; consumables should be able to be disposed of as biosafety waste; simple trash.	Increased biosafety of a novel test will enhance acceptability of the test by providers. Further information provided in WHO Laboratory Biosafety Manual [24].
Instrumentation	Instrument-free	Allow for separate specimen preparation device (e.g. mini-centrifuge)	The simpler, more portable and durable/robust the test is, the more likely it will be implemented in peripheral settings. Ideally an instrument free test (e.g. immunochromatographic test) would be the preferred optimal solution but this is likely not feasible with the analytical sensitivity that is necessary and a small specimen volume from a fingerstick.
Power requirements	If device necessary then: battery-operated with recharging solution (e.g. solar) and circuit protector lasting up to 3 days of constant use and able to run off standard electricity	Rechargeable battery or solar power lasting at least 8 h.	Continuous power is not always available at the level of a health and microscopy centre and even less likely at primary care clinics, therefore a battery-operated device with charge possibility conceivably through solar power would be most ideal in order for a test to fit into the entire breadth of settings [21, 23]
Maintenance/ calibration	Disposable, no maintenance or calibration required If device necessary then: preventative maintenance at 2 years or >5000 specimens; include maintenance alert; remote calibration	Preventative maintenance at 1 year or >1000 specimens; only simple tools/minimal expertise required; include maintenance alert. Swap-out of platforms permitted.	If a device is anticipated to have a longer lifespan, then a maintenance alert is essential to ensure proper functionality in settings where it is unlikely that the same person will always handle the device and records will be kept on duration of use. It is essential that only simple tools/minimal expertise are necessary to do the maintenance given that service visits are difficult outside of urban settings.
Data analysis	Integrated data analysis	Integrated data analysis (no requirement for PC); exported data capable of being analysed on a separate or networked PC.	
Connectivity	If device necessary then integrated connectivity; if no device necessary, then the test should allow data export via a separate reader. Full data export (on usage of device, error/invalid rates, and personalized, protected results data) over USB port and network. Network connectivity through Ethernet, WiFi, and/or GSM/UMTS mobile broadband modem. Results should be encoded using a documented standard (such as HL7) and be formatted as JSON text. JSON data should be transmitted through http(s) to a local or remote server as results are generated. Results should be locally stored and queued during network interruptions and sent as a batch when connectivity is restored.	Full data export (on usage of device, error/invalid rates, and personalized, protected results data) over USB port and network. Network connectivity through Ethernet, WiFi, and/or GSM/UMTS mobile broadband modem. Results should be encoded using a documented standard (such as HL7) and be formatted as JSON text. JSON data should be transmitted through http(s) to a local or remote server as results are generated. Results should be locally stored and queued during network interruptions and sent as a batch when connectivity is restored.	Data export will enhance surveillance, device and operator management and allow for supply chain management.
Result capture, documentation, data display	If instrument-free: ability to save results via separate reader. If device necessary: integrated results screen and ability to save and print results; USB port. On-instrument visual readout and the ability to add information (patient ID, operator ID, date location, etc.)	Ability to save results The test menu should be simple with integrated LCD screen; simple key pad or touch screen.	Results should be simple to interpret (positive/negative for HCV detection).
Operating temperature/ humidity/altitude	Between +5 to +40^o^ C at 90% humidity and at an altitude of 3000 m	Between +10^o^ to +35^o^ C at 70% humidity and at an altitude of 2000 m	High environmental temperatures and high humidity are often a problem in countries where HCV is endemic.
Reagent kit transport	No cold chain required; tolerance of transport stress for a minimum of 72 h at -15^o^ to +40 °C	No cold chain required; tolerance of transport stress for a minimum of 48 h at -15^o^ to +40^o^ C	Refrigerated transport is costly and often cannot be guaranteed during the entire transportation process. Frequent delays in transport are commonplace.
Reagent kit storage/stability	2 years at +5 °C to +40^o^ C at 90% humidity & transport stress (72 h at 50^o^ C); no cold chain required	12 months at +5 °C to 35^o^ C, 70% humidity, including transport stress (48 h at 50^o^ C); no cold chain required	High environmental temperatures and high humidity is often a problem in many countries where HCV is prevalent.
Internal process quality control	Internal full-process control, positive control & negative controls	External positive control	In addition to compatibility with existing external quality assessment schemes
Pricing			
Maximum price for individual test (reagent costs only; at scale; ex-works)	< US $ 5	< US $ 15	For a one-step solution, the cost needs to be low, as a trade-off in the ease-of-use/performance for price would not be accepted. Conversely, in a two-step solution, a higher cost is more likely to be accepted, as people would be willing to make a trade-off provided the overall cost of the algorithm remains low. Cost-benefit analyses are needed to explore different options. Trade-offs between optimal characteristics may be necessary to achieve optimal pricing. Preferences about acceptable trade-offs need to be further defined.
Maximum price for instrumentation	< US $ 2000	< US $ 20,000	The lower the price for instrumentation, the lower the up-front cost to a health-care system would be and thus the lower the barrier to implementation. Further modelling is necessary to confirm the maximal price estimated. Price should include warranties, service contracts and technical support. Alternatively, rental agreements for equipment should be an option.

The TPP was finalized using input from the Delphi-like survey and discussion at the consensus meeting (April 22, 2015, Vienna, Austria). TPPs were combined because characteristics were similar for both, independent of whether the test envisioned was a RNA or cAg-based test. The TPP needs to be considered in the context of different types of testing strategies (one-step versus two-step)

*Compared to a HCV RNA reference test performed on plasma

**Ex-works, including proprietary reagents and consumables cost only (without instrumentation), produced at scale

### Values and preferences survey of implementers and users of hepatitis C tests

In the development of the WHO 2017 guidelines on hepatitis B and C testing, the GRADE system (Grading of Recommendations, Assessment, Development and Evaluation) was used to categorise both the strength of recommendations as strong or conditional (based on consideration of the quality of evidence, balance of benefits and harms, acceptability or values and preferences of end-users, resource use and programmatic feasibility). To assess the values and preferences for trade-offs between different test characteristics in the TPP, different testing strategies and approaches, a four-part online survey tool was developed by FIND and WHO with input from MSF, CDC and Médecins du Monde using SurveyMonkey software (SurveyMonkey Inc., Palo Alto, California, USA) Ivanova et al [35]. Survey questions were designed as multiple-choice with comments sections where appropriate (see Additional file 1; only the questions relevant to further the understanding around user preferences in regards to the TPP for HCV diagnostics are listed). The survey was undertaken in September 2015, and the invitation to participate was sent via email to 306 people on the WHO Hepatitis database and included in the FIND and Forum newsletters, and included clinicians, patient organizations, civil society representatives, national hepatitis programme managers, policy-makers and diagnostics and pharmaceutical industry employees. Responses were exported into a Microsoft Excel table (Microsoft Office 2010, Microsoft Corporation). A descriptive analysis was performed using R statistical software (version 3.2, R Foundation for Statistical Computing). Responders were not required to answer to all questions, thus the denominator for each response are the number of responders to the specific question. Only findings relevant to user preferences in regards to the TPP for HCV diagnostics are presented here.

## Results

### Stakeholder participants in TPP development

**Fig. 2:**
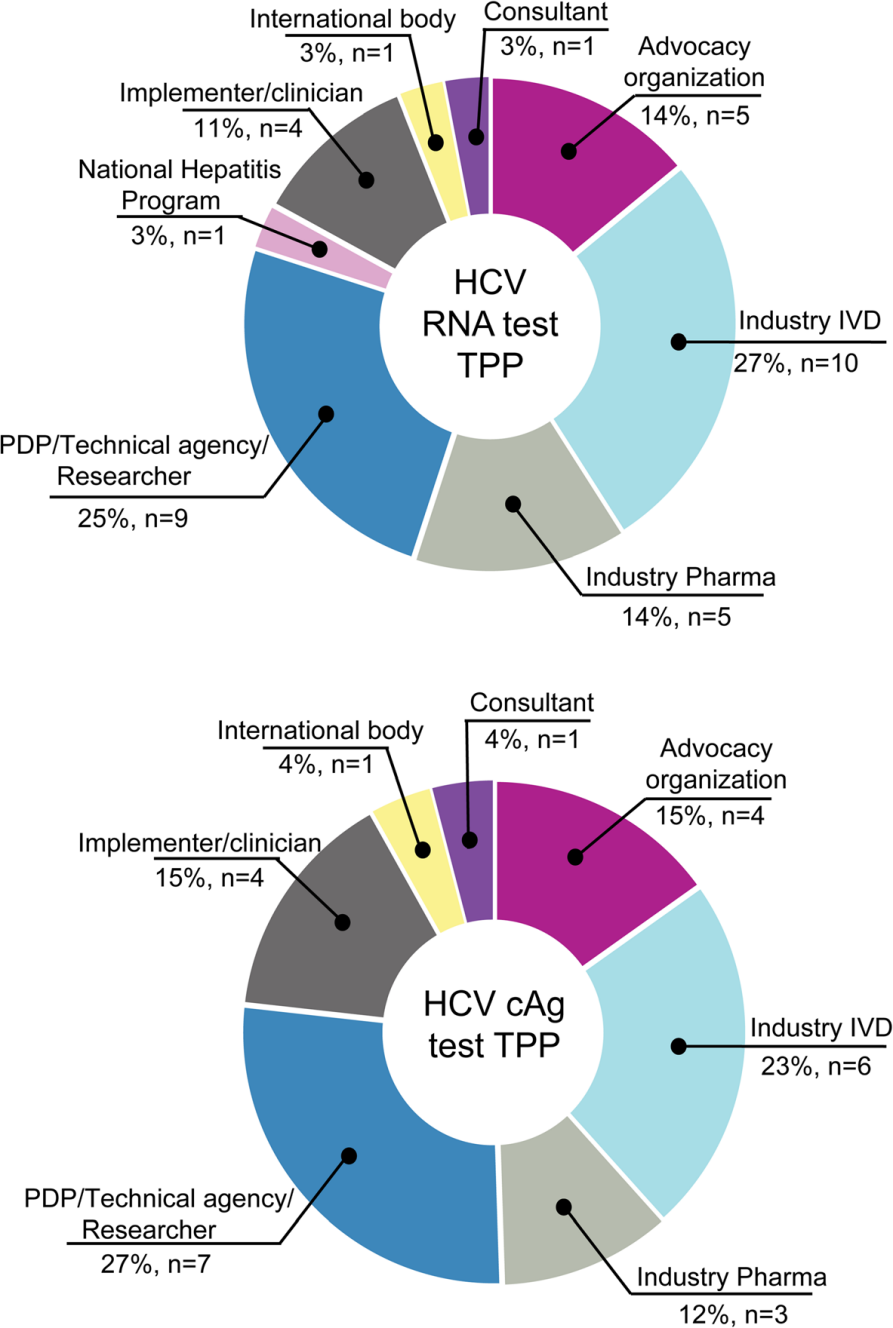
Information about respondents. Professional profiles of 36 respondents to the HCV RNA and cAg TPPs

Of the 50 organizations/individuals invited to participate in the Delphi-like TPP consultation, 36 responded to the HCV RNA TPP and 26 to the cAg TPP, and 35 attended the consensus meeting in Vienna. About half of responders were from the in vitro diagnostics industry or product development partnerships/technical agencies/researchers (27% and 25% respectively), 14% were from advocacy organizations and the same from the pharmaceutical industry, 11% were implementers/clinicians and the remainder (3% each) represented national hepatitis programmes, international bodies and consultants (Fig. 2).

### Consensus on TPP characteristics

The consensus process was used to define the characteristics within the four categories of the TPP: scope, performance, operational characteristics and price. Of the 27 characteristics across the four categories, there was greater than 50% agreement on all of the TPP characteristics. There was even greater than 75% agreement for all characteristics except the minimum clinical sensitivity and the price. All stakeholders agreed that the TPP for HCV RNA and for HCV cAg test could be combined into one TPP for a near-patient HCV virological test.

### Target specifications for key characteristics of TPP for HCV virological test

The final characteristics in the TPP are summarized in Table 1. Key characteristics are described below.

#### Scope of the test

##### Goal of the test

The near-patient test defined in the TPP is for diagnosis of active, viraemic HCV infection. Two testing strategies are considered: (i) a one-step strategy using a single HCV RNA or cAg test (ii) a two-step strategy using an HCV antibody screening test followed by a confirmatory HCV RNA or cAg test, if antibody positive (Fig. 1). Conceivably, provided the prevalence is substantial and the cost of the test is sufficiently low, it could be used in a one-step diagnostic strategy, thus obviating the need for an upfront antibody screening test. Additionally, since the risk of reinfection is substantial in some population groups, this means that certain high risk groups who have been treated for HCV infection in the past should be tested again for HCV RNA or HCV cAg directly as HCV antibody screening test is expected to be positive.

Furthermore, the test should be also usable to confirm cure upon treatment completion.

##### Target population

The target population for the test was considered to be all persons in countries with a medium to high prevalence of HCV (1.5–3.5% and >3.5%) as well as high-risk populations in low prevalence settings (<1.5%).

##### Target operator

The target operator of a test optimally was considered to be a community worker with minimal training particularly in the context of a one-step testing strategy, where easy-to-use testing close to the patient is paramount to increase access to care. At a minimum the test should be easy enough to perform by a health-care workers or laboratory technicians with limited training (i.e. the test would need to be integrated test with minimal additional and no precision steps; see operational characteristics below).

#### Performance characteristics

##### Analytical and clinical sensitivity

The analytical sensitivity or the lower limit of detection (LOD) describe the smallest concentration of an analyte that can be reliably measured by an analytical procedure. Current available HCV RNA assays are able to detect as low as 5 IU/ml [18]. Trade-offs such as cost and ease-of use have to be considered in setting targets for analytical sensitivity. If the optimal specimen type (i.e. capillary blood; see below) or a simplified transport tool (e.g. dried blood spots, DBS) were considered, a lower analytical sensitivity (<1000 IU/ml) would be technically difficult to achieve.

In the proposed TPP, the optimal analytical sensitivity was defined as 200 IU/mL for both HCV RNA and cAg tests, while the minimum acceptable level was 1000–3000 IU/mL. Translating the proposed minimal analytical sensitivity into clinical sensitivity (i.e. the probability that the test will correctly identify an infected individual), the LOD would result in >95% of viraemic patients correctly diagnosed based on available limited data on distribution of viral load measurements in those diagnosed with viraemic HCV infection [29, 30]. The majority of stakeholders felt that from a population perspective any test with 95% sensitivity that would substantially improve access to diagnosis and treatment would be a step forward. Further, it was suggested that an optimal sensitivity of 200 IU/ml would likely be sufficient to detect >99% of viraemic patients. Both limits of detection need to be validated in larger surveillance studies on quantitative viral load measurement.

##### Polyvalency

(i.e., same device can be used for different tests) Polyvalency was considered a key characteristic to increase affordability of and access to a diagnostic test by the stakeholder group. Given that polyvalency was initially not voted upon in the Delphi-like survey, a vote was taken in the consensus-meeting. More than three-quarters of stakeholders supported the development of polyvalent platforms that integrate diagnosis of multiple diseases such as HIV, TB and hepatitis B with HCV as an optimal characteristic. Multiplexing (i.e. detecting different analytes in a single sample at the same time) was seen as advantageous for some tests by but not considered as essential by the majority.

##### Quantitation

The majority of stakeholders (>85%) thought that a quantitative test is no longer required if DAA regimens are used, as a decision to treat and confirmation of cure is based on presence of viraemia, and response-guided therapy is likely no longer required or feasible [31]. However, several stakeholders highlighted that a quantitative test is still advantageous to address specific research questions. Furthermore, diagnostic manufacturers suggested that there is only a small cost differential for the development of a qualitative and quantitative test. Therefore, quantitation was integrated as an optimal target.

#### Operational characteristics

##### Specimen type

All stakeholders (100%) agreed that the optimal test is the one that can be performed on capillary whole blood without any requirement for any additional laboratory equipment as the need for phlebotomy to draw venous whole blood would limit the applicability of the test in lower settings of care. If plasma is to be a specimen type (minimal criteria), the plasma separation step should be integrated into the instrument.

##### Specimen preparation

For the specimen preparation, the majority (>86%) of stakeholders suggested that an integrated specimen preparation (including plasma separation if needed) would be optimal. At a minimum, less than 2 steps should be required (with no steps being precision volume control and precision time steps). These requirements are in line with the skills set a test operator would be expected to have at the settings of intended use [32].

##### Time to result/throughput

The optimal turnaround time defined in the TPP is less than 15 minutes and, at a minimum, the test results should be delivered within 1 hour. The need for a rapid turn-around time, the possibility for batching and/or random access for testing, and the testing of multiple specimens at the same time are interrelated. The time to result is probably the most important parameter, as extending the wait time for patients will possibly result in loss to follow-up [33, 34]. The ideal time to result might vary largely between settings where the patient is tested and has been further evaluated in the values and preferences survey. In order to be deployable as a test for point-of-care, the result should be available within the same visit. Most current immune-chromatographic rapid tests produce results within 20 minutes.

##### Instrumentation and power requirements

The ideal test was considered to be an instrument-free test. If an instrument is necessary, then it needs to be battery operated, ideally with a recharging solution (e.g. solar) and circuit protector lasting up to 3 days of constant use.

For the description of other performance and operational characteristics please refer to the Table 1.

#### Price of the test

The specification on the ex-works test price (i.e. price of test at the manufacturing factory) at scale had the lowest level of agreement among stakeholders (only 58% agreement for minimal price for HCV RNA test at <US $ 15 and 73% for HCV cAg test at <US $ 10), while 75% agreed on an optimal cost of an HCV RNA of <7 $ US and 85% agreed on an optimal cost of a HCV cAg test at <US $ 3. The opinions on the minimal cost were split between stakeholders, with many considering the price was too low and others the price was too high. During the discussion, the main factors raised by the stakeholders that would influence the price of tests included the following: (i) the complexity of the test, for example, if there was complex specimen preparation required for the release and detection of HCV cAg; and (ii) the volume of tests undertaken, which was expected to be lower initially given the anticipated lower demand for HCV diagnostics, as compared to diagnostics for other diseases such as HIV due to the lack of hepatitis C care services in LMICs. Furthermore, stakeholders highlighted that the ex-works price, while better compared to other diagnostics, would not be the price seen by the end-user as shipping costs, import taxes, customs charges and local distribution costs would be added on. The control and limitation of these add-on costs was considered paramount to enable the scale up of testing. Ultimately the cost was set at US $ 5 optimally and US $ 15 minimally and the majority of stakeholders (>75%) agreed under the precondition that substantial trade-offs would have to be accepted (e.g. in regards to sensitivity) with a test at US $ 5.

### Values and preferences (V&P) survey

The V&P survey questionnaire covered current hepatitis B and C testing practices and preferences for future testing diagnostics. Only the findings relevant to further the understanding around user preferences in regards to the TPP for HCV diagnostics are presented here (see Additional file 1), while the major part of the survey results is reported separately [35].

In total, 104 respondents from 43 countries (56 respondents from 20 high-income countries and 48 respondents from 23 LMIC) participated in the survey. The absolute majority of respondents (94 out of 104) were implementers (i.e. people working in projects for HCV care in countries either within governmental or non-governmental organizations) and IVD industry employees accounted for only 5.8% (6/104). More than a half of the respondents (65 out of 104) had more than 10 years of experience in the field of viral hepatitis.

**Fig. 3:**
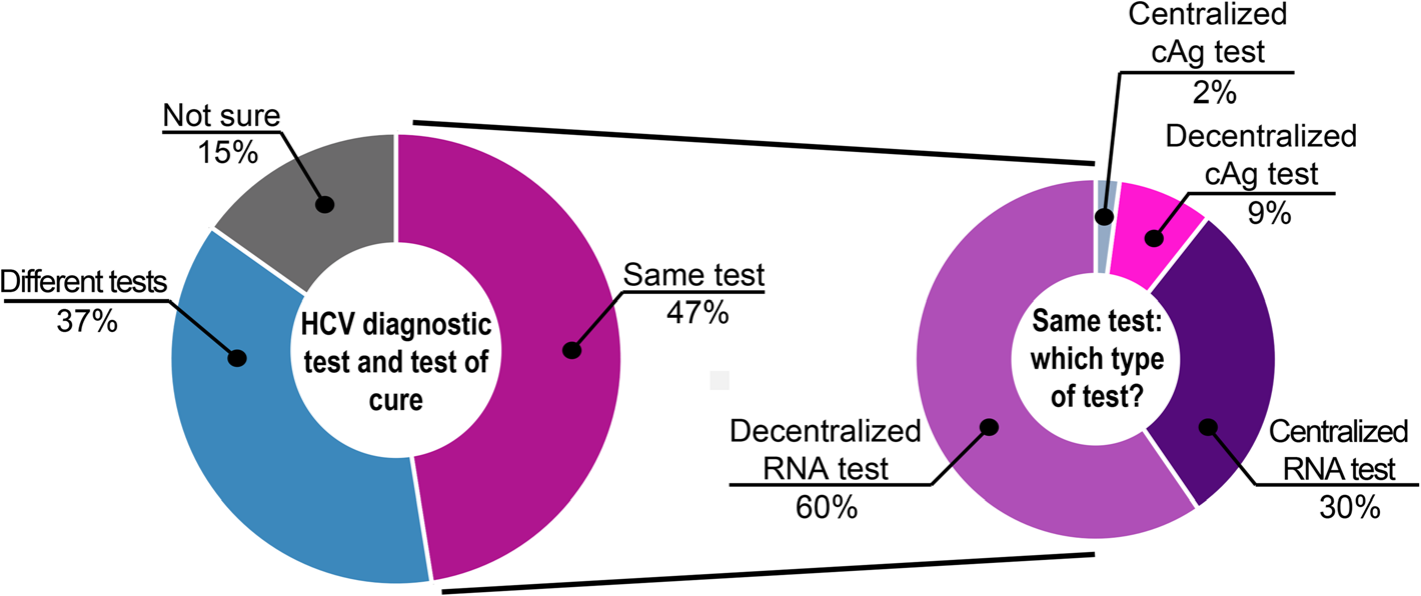
Preference for the HCV diagnostic test and the test of cure to be the same or different. Almost half of respondents preferred that the diagnostic test and test of cure be the same and about two thirds of these preferred that it be a decentralized (i.e. near-patient) HCV RNA test

Responses to survey questions on several key TPP characteristics were as follows:

*Test of cure:* Almost half of the respondents (47 out of 99) considered that the diagnostic test for HCV viraemic diagnosis should the same as the one for test of cure (Fig. 3). Of these forty-seven, 90% preferred a HCV RNA test (decentralized or laboratory-based) primarily because of its high accuracy over a cAg test.

To explore preferences for future HCV diagnostics in resource-limited settings, the survey specifically collected opinions of stakeholders from LMIC on desired performance and operational characteristics of future HCV test and acceptable trade-offs.

**Fig. 4:**
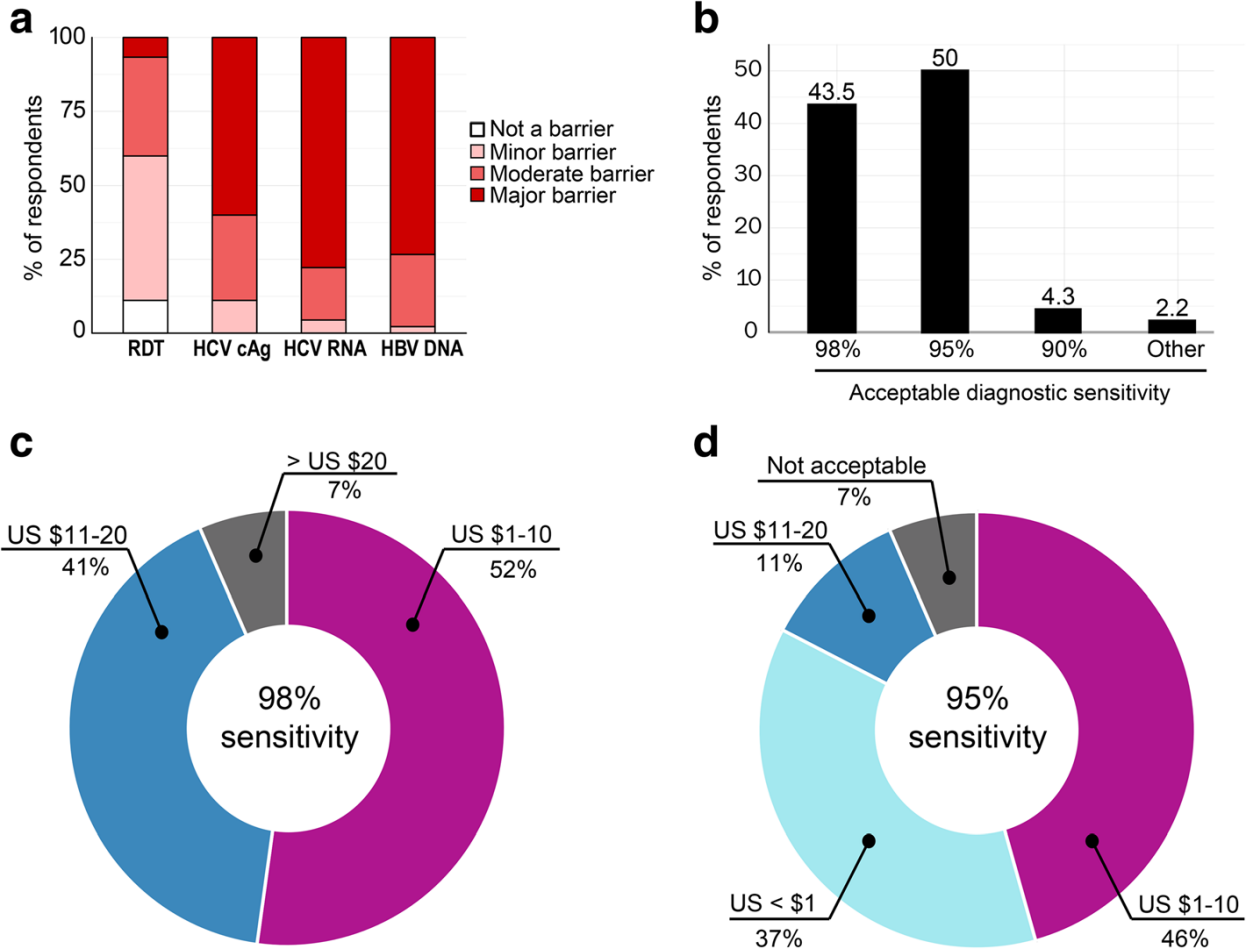
Preferred sensitivity and acceptable trade-offs between sensitivity and price. Over 50% of respondents see current price of HBV DNA, HCV RNA, and to a lesser extent HCV cAg, is seen as a major barrier to scale up the testing **a**. Half of respondents consider that 95% as an acceptable diagnostic sensitivity for a one-step diagnostic test whereas 43% prefer 98% **b**. Respondents were willing to pay more for a higher sensitivity and were willing to compromise on sensitivity for a cheap test **c** and **d**

*Sensitivity:* Half of the respondents (23 out of 46 LMIC respondents) considered that 95% was the lowest acceptable level of clinical sensitivity (Fig. 4b), and 43% (20 out of 46) preferred a sensitivity of 98%. However, when asked about the price for tests with different sensitivity, most of the respondents (83%) indicated that a test with 95% sensitivity is acceptable if the price is lower than US $ 10 (Fig. 4c and d). Only 7% (3 out of 46) felt that a 95% sensitivity would be unacceptably low.

**Fig. 5:**
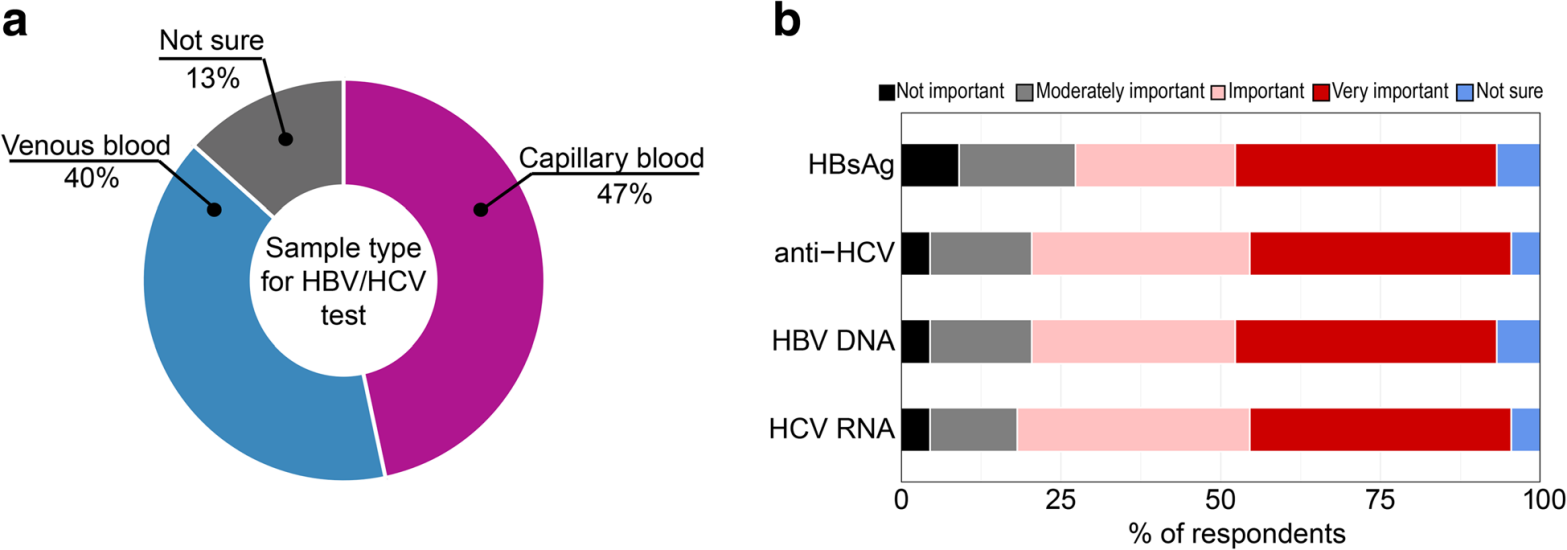
The importance of specimen type and the ability to use dried blood spots. Capillary and venous blood are the preferred specimen types **a** and most respondents consider the ability to use DBS as a specimen type important or very important **b**

*Specimen type:* Half of respondents (21 out of 45) expressed a preference for a test that can be performed on capillary blood even if this was at the expense of sensitivity (Fig. 5a). The majority (75%) also considered the ability of the test to use dried blood spots (DBS) as important or very important particularly for centralized testing (Fig. 5b).

**Fig. 6:**
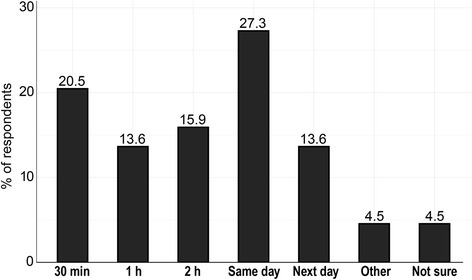
Acceptable turn around times between taking the specimen and returning the result to the service provider. A maximum of a same day result is preferred

*Turnaround time:* 21% of respondents considered 30 minutes the optimal turnaround time, while 14% and 16% were willing to accept 1 or 2 hours, respectively. Twenty-seven percent were willing to accept a turnaround time of 1 day and 14% allowed even for the next day (Fig. 6).

*Price:* The majority of respondents considered current prices of virological tests as a barrier to implementation, particularly for HCV RNA tests (77%) and less so for cAg tests (60%) (Fig. 4a). When asked about a maximum acceptable price per test (ex-works and at scale), 52% (24 out of 46) of respondents indicated US $ 1–10 for a test with 98% sensitivity, while 41% (19 out of 46) were ready to pay up to US $ 20 (Fig. 4c). Half of respondents (21 out of 46) would also accept a 95% sensitive test at the cost of US $ 1–10, while 37% (17 out of 46) considered that such a test should cost < US $ 1 (Fig. 4d).

**Fig. 7:**
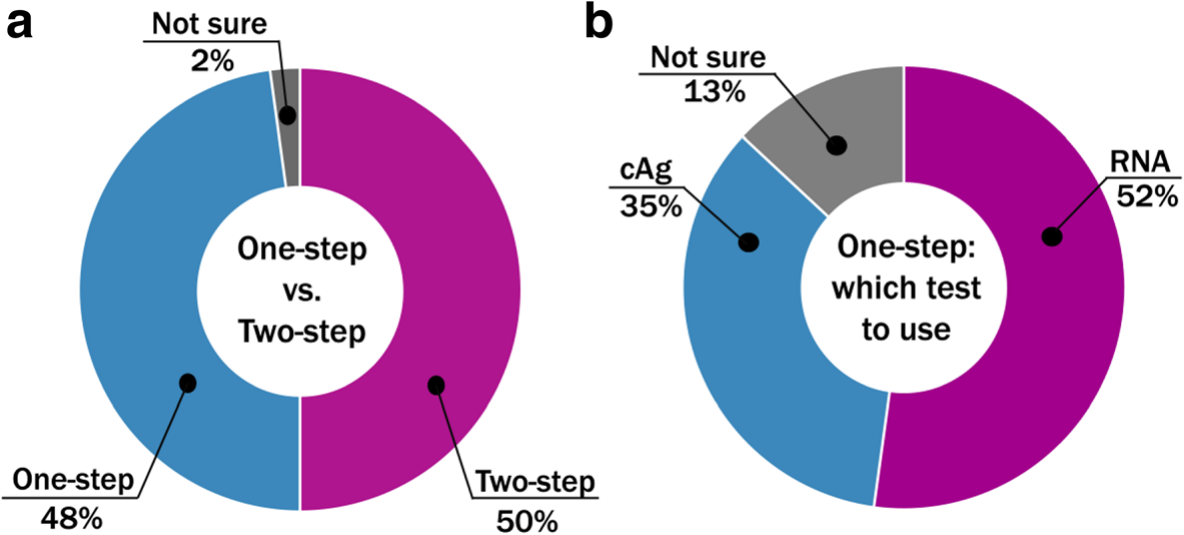
Preferences for HCV testing strategy. Half of respondents consider two-step testing strategy (see Figure 1) to be an optimal future testing approach in LMIC, while the other half prefer currently used two-step approach **a**. If one-step testing strategy is implemented, more than a half of respondents prefer to use HCV RNA test **b**

*Testing strategy:* When asked about preferred testing strategy (Fig. 1), half of respondents (50%) considered a two-step approach with an HCV serological test followed by confirmatory HCV virological test to be an optimal testing strategy. Almost the same number of respondents (48%) expressed preference for a one-step testing strategy that implies a single HCV virological POC. They explained their preference by the simplicity of the algorithm and reduced rate of loss-to-follow-up (Fig. 7a). If a one-step strategy is used, 52% of the respondents (24 out of 46) preferred an HCV RNA test as a single virological test, while 35% (16 out of 46) considered that an HCV cAg test would be an optimal diagnostic solution (Fig. 7b).

## Discussion and conclusions

For the next generation of near-patient/point-of-care diagnostic tests for HCV viraemia based on HCV RNA or HCV cAg detection to succeed in improving access to care, certain key specifications need to be met. We report on these key specifications for scope, performance, operational characteristics and price in a TPP utilizing a consensus-based process with key global stakeholders. The most critical features of the assay identified are sensitivity, specimen type, steps required to perform the test and price. The additional V&P survey that was performed to understand the values and preferences of end users and implementers around specifications set in the TPP, highlights potential compromises on performance features to achieve lower cost and ease-of-use.

The TPP sets high requirements for optimal test accuracy (>99%) but set a lower target under minimal criteria (90–95%). The V&P survey showed that a majority of end-users and implementers considered a test with 95% sensitivity to be acceptable while 90% sensitivity was considered to be insufficient. Modelling studies are required in order to determine the minimum sensitivity that is needed for the point-of-care test to lead to the substantial improvement in HCV detection on a population level. Also, only limited data are available to understand the limit of detection that corresponds to 90–95% clinical sensitivity. Available data show that the majority of infected individuals with chronic HCV infection have viral loads between 10^4^ and 10^7^ IU/mL, however a transient dip to values below 10^3^ IU/ml can be observed in patients with partial viral control within the first 6 months [29]. Thus, the optimal limit of detection of 200 IU/mL set in the proposed TPP should detect almost all viraemic patients (i.e. >99%(LOD) i.e. optimal). However, the data are limited in terms of capturing different populations, stages of disease and genotypes and further comprehensive studies are needed to confirm the acceptable LOD. The V&P survey indicated that many stakeholders would be willing to compromise on sensitivity if such a test could be achieved at decreased cost and increased ease-of-use. Certainly, test design decisions such as the use of capillary blood for a specimen will limit the ability to achieve a LOD below 1000 IU/mL. The detection of cAg instead of RNA will also limit the ability to reach an optimal analytical sensitivity, as even laboratory-based assays that include complex specimen preparation (i.e. Abbott Architect) can detect femtomolar amounts of cAg and have a LOD equivalent to about 3000 IU/ml for an RNA assay.

The TPP also sets challenging operational characteristics to ensure the usability of the test in low levels of the healthcare system, close to where the patients first present. Capillary blood is defined as an optimal specimen type as it greatly reduces the need for skilled workers, precision pipetting, centrifugation or nearby complex laboratory infrastructure, which is particularly beneficial at lower levels of the healthcare system where these resources and skills are lacking [32, 34]. The acceptable and optimal turnaround time will enable diagnostic decision making possibly on the same day, reduce costs of transportation for patients and so in turn loss to follow-up [33]. However, results of the V&P survey suggest that an even longer turn-around time would be acceptable in some settings. Other operational characteristics, such as the requirement for an instrument to run of battery power, the need for stability in wide ranges of temperature and humidity reflect upon the possible settings the test could be used in LMIC.

A further consensus conclusion is that on-treatment monitoring does not appear to be necessary or useful with DAA regimens as the early reduction in viral load does not correlate with cure [31]. However a test of cure is still important. Given the high correlation between HCV cAg and HCV RNA levels [36], either test would likely be suitable as a test-of-cure. Again, limited data is available on the required analytical sensitivity for a test of cure. Cohn et al. have suggested that 3000 IU/mL is likely suffice for this indication [37], however further data are needed about the rapidity and trajectory of a viral load rebound after treatment failure across different populations, fibrosis stages and genotypes particularly with new DAA regimens. The WHO guidelines recommend a single test of cure performed either at 12 weeks or 24 weeks after the end of treatment [1].

The topic on which unsurprisingly there was the least consensus between stakeholders in the meeting as well as in the V&P survey was the price per test, with end-users and manufactures disagreeing on acceptable prices. A key conclusion was the need for greater transparency, both from the manufacturers themselves on cost of goods and volume-based price-breaks, but also from implementers on cost of distribution so that costs can be disaggregated and end-users can gauge what a fair price should be for the goods purchased. Approaches to reducing costs include (i) countries and donors coming together to pool procurement and forecasting; (ii) launch of competitive tenders; (iii) request bundled pricing on the back of HIV and TB test roll-out; (iv) exploration of different supply chain models for LMIC to reduce the distribution costs incurred; and (v) integrate HCV care within programs for other diseases, such as HIV, TB and hepatitis B, to leverage investments made in polyvalent platforms and care infrastructure. Particularly for the pooled procurement and forecasting there are many lessons learned from the HIV experience.

The strengths of this work include:- (i) it followed a formalized step-wise process established for the development of consensus-based TPPs prior; (ii) It included a broad group of stakeholders; (iii) the TPP process was complementary by a survey that was able to elucidate trade-offs between characteristics. However, we also have to acknowledge limitations. The TPP reflects the opinion of the stakeholders represented in the Delphi-like survey and at the meeting. While we attempted to have a large group of stakeholders that was representative of all the different groups (national Hepatitis programs; implementers and clinicians (including from NGOs); technical agencies and researchers; international organizations; advocacy organizations/civil society; industry (pharmaceutical and IVD-manufacturers), not everybody was able to participate. It also has to be noted that industry representatives accounted for a large percentage of the stakeholders however an analysis of the TPP survey excluding the responses from industry representatives showed minimal differences to an overall analysis (data not shown).

As part of the discussion the following key research questions were identified, that should be addressed to inform future test development and guidelines:

1. Improve understanding on what clinical sensitivity would be achieved with an optimal analytical sensitivity of 200 IU/ml and a minimum analytical sensitivity of 1000–3000 IU/ml. Also, what are the characteristics of HCV-infected individuals with a viraemia of <1000–3000 IU/ml who are missed by an assay with minimum sensitivity? Are they less prone to develop substantial HCV disease? Are they more likely to resolve their infection?
2. What should be the sensitivity of a test of cure and when should such a test be best performed to optimize feasibility, ideally with the same platform as used for detection and minimize loss-to-follow up.
3. What is the cost effectiveness of a one-step approach versus a conventional two-step approach (with HCV serological test first followed by HCV RNA or cAg test) in different prevalence settings?

The ability to diagnose HCV infection in LMIC near to the patient or at the point of care is an important aspect of scaling up access to HCV care. While centralized platforms are already available, an accurate, easy-to-use and affordable diagnostic solution to confirm active HCV infection near to the patient is lacking and urgently needed in LMIC. Independently, such a test would likely be of use in expanding diagnostic testing in resource-rich settings. In considering the needs in resource limited settings, the stakeholder community and donors need to come together to now translate this high priority TPP into actual tests that can help to curb the HCV epidemic and ensure affordable and accessible diagnosis for HCV.

## Additional file

Additional file 1: The V&P survey questionnaire. One table in Microsoft Word format that where survey questions are listed in the left column and answer options in the right. Only the questions linked to data presented here are listed. (DOCX 21 kb)
