# Supplementary material for: Optimising diagnosis of viraemic hepatitis C infection: the development of a target product profile
Source: BMC Infect Dis. 2017 Nov 1;17(Suppl 1):707. doi: 10.1186/s12879-017-2770-5 (PMC5688443; doi:10.1186/s12879-017-2770-5)
Supplement: Additional file 1: — The V&P survey questionnaire. One table in Microsoft Word format that where survey questions are listed in the left column and answer options in the right. Only the questions linked to data presented here are listed. (DOCX 21 kb) [file 12879_2017_2770_MOESM1_ESM.docx]

**Additional file 1**

**Table : The V&P survey questionnaire*.**

| **Question** | **Answer options** |
| --- | --- |
| Country | Please select one country for which you are most qualified to discuss current practices for HCV and HBV testing. |
| What is your role in relation to hepatitis testing? Check all that apply. | ☐ Medical doctor/ clinical officer  ☐ Medical assistant  ☐ Primary care provider  ☐ Laboratory expert  ☐ Researcher  ☐ In vitro diagnostics industry personnel  ☐ Employee/Consultant of a national or international NGO  ☐ Employee of an international organization (e.g., WHO)  ☐ National programme administrator  ☐ Programme implementer  ☐ Policy maker  ☐ Activist  ☐ Other (please specify) |
| How long have you been working in the field of viral hepatitis? | ☐ Less than 1 year  ☐ 1-2 years  ☐ 3-5 years  ☐ 5-10 years  ☐ More than 10 years |
| What is the broadest area in which you are familiar with the HBV and HCV testing that is available? | ☐ The health programme I work in  ☐ Health district  ☐ Province/state  ☐ National level  ☐ I am not familiar with testing  ☐ Other (please specify) |
| 14. Of the following algorithms for HCV diagnosis, which one would you prefer:  • **Two-step testing**: a screening rapid test (for HCV antibody) followed by a laboratory-based confirmatory test (for HCV RNA or core antigen)  *or*  • **One-step testing**: a low-cost (less than US$ 15), point-of-care HCV virological test (for HCV RNA or core antigen) | ☐ One-step  ☐ Two-step  ☐ Not sure/need more information |
| Under the previously proposed two-step testing algorithm, the existing confirmatory test for HCV RNA has a diagnostic sensitivity of >99% (i.e. less than 1% of test results are false negatives), while the alternative, a HCV core antigen test, costs less and could increase access to HCV diagnosis, but has a diagnostic sensitivity of 95% (i.e. 5% false negative). Considering this, which type of test would you prefer as a one-step point-of-care diagnostic test? | ☐ HCV RNA test  ☐ HCV cAg test  ☐ Not sure/need more information |
| What would you consider to be a maximum acceptable manufacturer’s price per test (i.e. price that does not include delivery or import costs) for a point-of-care solution that could be used in a one-step algorithm?   1. For an HCV RNA test that could be used in a one-step algorithm with 1-2% false negatives: 2. For an HCV core antigen test that could be used in a one-step algorithm with 5% false negatives: | ☐ More than US$ 20  ☐ US$ 11-20  ☐ US$ 1-10  ☐ Less than US$ 1  ☐ More than US$ 20  ☐ US$ 11-20  ☐ US$ 1-10  ☐ Less than US$ 1  ☐ Such a test is not acceptable |
| What would be the lowest acceptable sensitivity for a one-step HCV testing algorithm in a point-of-care setting? | ☐ 98% (i.e. 2% false negatives)  ☐ 95% (i.e. 5% false negatives)  ☐ 90% (i.e. 10% false negatives)  ☐ 85% (i.e. 15% false negatives)  ☐Other (please specify) |
| In your opinion, how much of a barrier is the actual price (the market price that includes test cost, delivery cost and margins) for each of the following tests (you can choose the same answer for more than one test):   1. Rapid Diagnostic Test for HCV antibodies/HBV antigen (HBsAg) 2. HCV Core antigen test 3. HCV RNA test 4. HBV DNA test | ☐ Major barrier  ☐ Moderate barrier  ☐ Minor barrier  ☐ Not a barrier |
| The viral hepatitis test performed from capillary (fingerstick) blood may result in a decrease in sensitivity as compared to venous blood (which requires a blood draw by a phlebotomist). However, capillary sampling is easier for point-of-care testing. Given these trade-offs, which blood specimen would you prefer for HBV/HCV diagnosis? | ☐ Capillary  ☐ Venous  ☐ Not sure/need more information  What is your motivation for your response? |
| Some tests are performed on plasma (a component of whole blood) instead of whole blood. How important is it to have the plasma separation integrated into the test device? | ☐ Very important  ☐ Important  ☐ Moderately important  ☐ Not important  ☐ Not sure/need more information |
| In your opinion, how important is it to be able to perform viral hepatitis testing using dried blood spots (i.e. blood samples can be collected and dried on a special card in point-of-care settings and then sent to a centralized laboratory for analysis)?   1. DBS testing for HBV antigen (HBsAg) and HCV antibodies 2. DBS testing for HBV DNA and HCV RNA | ☐ Very important  ☐ Important  ☐ Moderately important  ☐ Not important  ☐ Not sure/need more information |
| How long should be the maximum acceptable interval between taking the sample and returning the HCV test result in order to maximize the potential health impact? | ☐ 30 minutes  ☐ 1 hour  ☐ 2 hours  ☐ Longer than 2 hours but available the same day  ☐ Results available the next day or later  ☐ Other, please specify  Please explain your preference |
| Although having one test device for both HCV diagnosis and the test of cure is cheaper and easier to perform, it limits the settings in which they can be used. Having two different tests would allow the health system to use them in different settings (e.g. a low-cost one-step point-of-care test for HCV diagnosis and “gold standard” laboratory-based HCV RNA test as the test of cure). Please indicate which of the following would be most beneficial in your area. | ☐ Same test: HCV RNA test in a centralized setting  ☐ Same test: HCV core antigen test in a centralized setting  ☐ Same test: HCV RNA in a decentralized setting  ☐ Same test: HCV core antigen test in a decentralized setting  ☐ Different tests: a decentralized HCV RNA / Core antigen test for diagnosis and centralized HCV RNA test for treatment response monitoring  ☐ Not sure/Need more info to express preference |
| Longer timing between end of HCV treatment and test of cure could result in greater loss to follow-up. However, performing the test of cure too soon after treatment can result in false negative tests.  Keeping this in mind, how long do you think you could reasonably wait before performing a test of cure without increasing your rate of loss to follow-up? | ☐4 weeks  ☐8 weeks  ☐12 weeks  ☐24 weeks  ☐No need for the test of cure after direct-acting therapy  ☐Not sure  Please explain your preference |

*****Only questions connected with data reported here are presented
